# Anti-MDA5 Amyopathic Dermatomyositis—A Diagnostic and Therapeutic Challenge

**DOI:** 10.3390/life12081108

**Published:** 2022-07-23

**Authors:** Anca Bobirca, Cristina Alexandru, Anca Emanuela Musetescu, Florin Bobirca, Anca Teodora Florescu, Magdalena Constantin, Tiberiu Tebeica, Alesandra Florescu, Sebastian Isac, Mihai Bojinca, Ioan Ancuta

**Affiliations:** 1 Department of Internal Medicine and Rheumatology, Carol Davila University of Medicine and Pharmacy, 050474 Bucharest, Romania; anca.bobirca@umfcd.ro (A.B.); mihai.bojinca@umfcd.ro (M.B.); ioan.ancuta@umfcd.ro (I.A.); 2 Department of Internal Medicine and Rheumatology, “Dr. Ion Cantacuzino” Clinical Hospital, 011437 Bucharest, Romania; maria-cristina.steopoaie@rez.umfcd.ro (C.A.); anca-teodora.florescu@rez.umfcd.ro (A.T.F.); 3 Department of Rheumatology, University of Medicine and Pharmacy of Craiova, 200349 Craiova, Romania; anca.musetescu@umfcv.ro (A.E.M.); alesandra.florescu@umfcv.ro (A.F.); 4 Department of General Surgery, Carol Davila University of Medicine and Pharmacy, “Dr. Ion Cantacuzino” Clinical Hospital, 050474 Bucharest, Romania; 5 2nd Department of Dermatology, Colentina Clinical Hospital, “Carol Davila” University of Medicine and Pharmacy, 020125 Bucharest, Romania; drmagdadinu@yahoo.com; 6 Department of Histopathology, “Dr. Leventer Centre”, 011216 Bucharest, Romania; tiberiutebeica@drleventercentre.com; 7 Department of Physiology and Neuroscience, “Carol Davila” University of Medicine and Pharmacy, 050474 Bucharest, Romania; sebastian.isac@umfcd.ro

**Keywords:** amyopathic dermatomyositis, anti-MDA5 antibodies, rapid-progressive interstitial-lung disease, COVID-19

## Abstract

Clinically amyopathic Dermatomyositis (CADM) is a rare subtype of idiopathic inflammatory myositis, associated with no muscular manifestations, which is more frequent in Asian women. Anti-melanoma differentiation-associated gene 5 (MDA5) antibodies are a recently discovered type of specific autoantibodies associated with myositis. The anti-MDA5 DM was initially described in Japan and later it was discovered that the target antigen was a protein implicated in the innate immune response against viruses, that is encoded by the melanoma differentiation-associated gene 5. Anti-MDA5 DM is characteristically associated with distinguished mucocutaneus and systemic manifestations, including skin ulcerations, palmar papules, arthritis, and interstitial-lung disease. Patients with anti-MDA5 positivity have a high risk of developing rapid progressive interstitial-lung disease (RP-ILD), with a poor outcome. As a result, despite high mortality, diagnosis is often delayed, necessitating increased awareness of this possible condition. Despite a severe course of lung disease and an increased mortality rate, there is currently no standard treatment. Recent insights based on observational studies and case reports support combined therapy with immunosuppressive drugs and corticotherapy, as soon as the symptoms appear. The aim of this paper is to describe anti-MDA5 DM, focusing on the recent literature about the unique clinical manifestations and therapeutic options, starting from a severe clinical case diagnosed in our Rheumatology Department.

## 1. Introduction

Dermatomyositis (DM) is a rare inflammatory myopathy characterized by muscular and skin lesions and a variety of systematic manifestations. This condition belongs to the heterogeneous group of idiopathic inflammatory myositis affecting symmetrical, proximal skeletal muscle with muscle inflammation and extra-muscular manifestations, such as skin rash, malignancy, cardiomyopathy, and interstitial lung disease (ILD) [1]. Autoantibodies are associated with the clinical manifestations of myositis and play a major role in the development of myopathy. There are two important subsets of autoantibodies: the myositis-specific antibodies (MSAs) and the myositis-associated antibodies (MAAs), which can be found in other connective tissue diseases. It is proven that some MSAs, especially anti-melanoma differentiation-associated gene 5 (MDA5), are associated with a high prevalence of ILD [2,3].

There are many subtypes of dermatomyositis, namely, classic DM, juvenile or adult type, clinically amyopathic DM (CADM) and cancer-associated DM. Clinically, amyopathic DM is a form in which the patients typically develop a skin rash but have little or no muscular involvement, and it is more frequent in Asian women in their fifth and sixth decades of life [4]. A more recently discovered subtype of this disease is anti-MDA5 DM, characterized by rapidly progressive Interstitial Lung Disease (RP-ILD) [5]. The prevalence of the Anti-MDA5 DM is dependent on the region, with the prevalence in European studies ranging from 1.3 to 10%, while in Japanese and Chinese cohorts it is higher (15–36.6%) [6,7]. The anti-MDA5 DM was initially described in Japan, and later it was discovered that the target antigen was a protein implicated in the innate immune response against viruses that is encoded by the melanoma differentiation-associated gene 5 [5,8].

In a recent study, anti-MDA5 autoantibodies were found exclusively in DM patients, being strongly associated with skin rashes, especially Gottron’s papules and heliotrope rash, and with ILD [6].

In this paper we aim to present a particular case of anti-MDA5 DM and provide some insights into the unique clinical manifestations and new therapeutic options.

## 2. Clinical Experience—Case Report

This paper presents the case of a 74-year-old Caucasian woman diagnosed with psoriatic arthritis one and a half year ago, according to skin biopsy (psoriasis) and peripheral arthritis and who was admitted to our hospital for a 6-month history of weight loss (~10 kilos), altered overall condition, nausea, vomiting and symmetrical polyarthralgia, especially affecting the small joints of the hands. For the psoriatic arthritis she received treatment with Methotrexate 15 mg weekly, for 8 months and then switched to leflunomide 20 mg daily, due to gastrointestinal intolerance. She decided on her own to discontinue the immunosuppressive therapy 7 months before the present hospitalization. She has never smoked and has never been exposed to a toxic environment.

**Figure 1:**
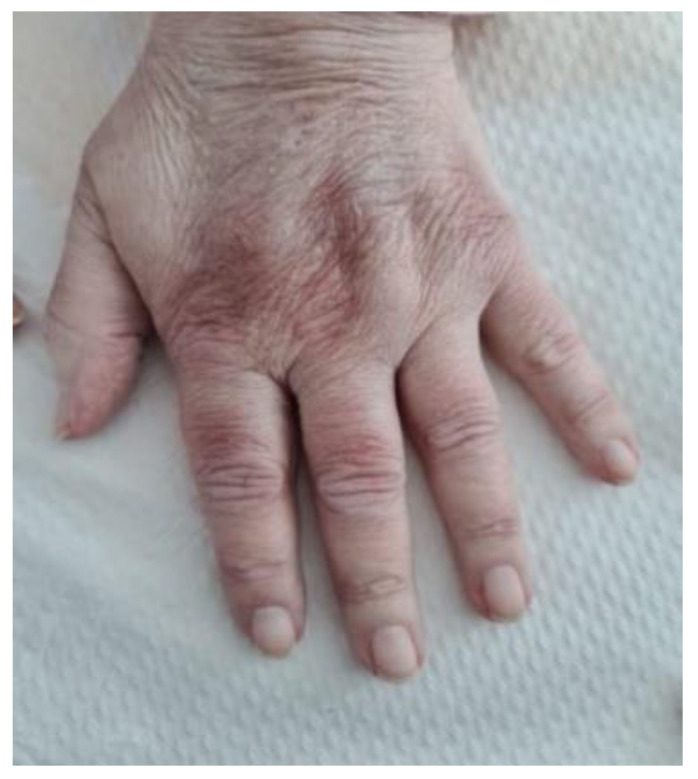
Hand of the patient; Gottron’s signs on the dorsum of the hand (mild manifestation).

The physical exam emphasized wheezing, crackling rales in both of the lower pulmonary fields, with an oxygen saturation level of 97% under ambient air and cutaneous lesions, namely xerotic eczema, mild periocular hyperpigmentation (heliotrope sign), and discreet Gottron signs on the dorsum of the bilateral metacarpophalangeal joints (MCP) and proximal interphalangeal joints (PIP) (Figure 1). She had no muscle weakness and she complained of 15 painful joints, predominantly the small joints of the hands, with no swelling.

The laboratory tests were performed, and the complete blood count test showed a mild normocytic, normochromic anemia, an important inflammatory reaction expressed by high values of erythrocyte sedimentation rate, (ESR- value 53 mm/h—normal value (NV) <30 mm/1 h), C-reactive protein (CRP-12X NV) and a slight elevation of hepatic enzymes and lactate dehydrogenase levels. Her D-dimer levels were 6.27 µg/mL (NV < 0.5) and serum ferritin level over 2000 ng/mL (normal range 15–150). The patient had mild hypocomplementemia, elevated antinuclear antibody (ANA) and negative tests for Rheumatoid Factor and Anticitrullinated Protein Antibodies. The creatine kinase (CK) and the renal function were normal, and she tested negative for hepatitis B and C. In the existing pandemic context, the PCR and autoantibodies for SARS-CoV-2 were negative.

**Figure 2:**
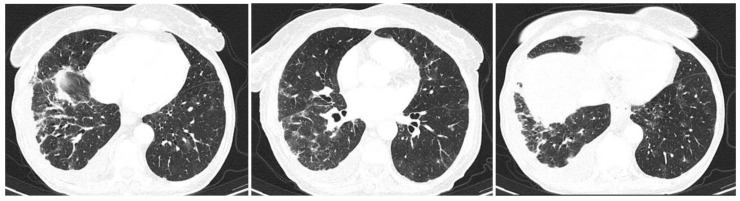
Chest CT scans one month prior to admission showing bilateral ground glass opacities of the lower lobes.

The patient had a whole body computed tomographic (CT) scan one month prior to our hospital admission, which revealed interstitial lung disease with interstitial infiltrates, ground glass opacities and traction bronchiectasis affecting predominantly the lower lobes of the lung (30% of pulmonary parenchymal architecture), with bilateral, mild pleural effusion (Figure 2), without any other abnormal findings regarding abdominal and pelvic regions. Having no respiratory symptoms, she was never evaluated in a pneumology unit.

**Figure 3:**
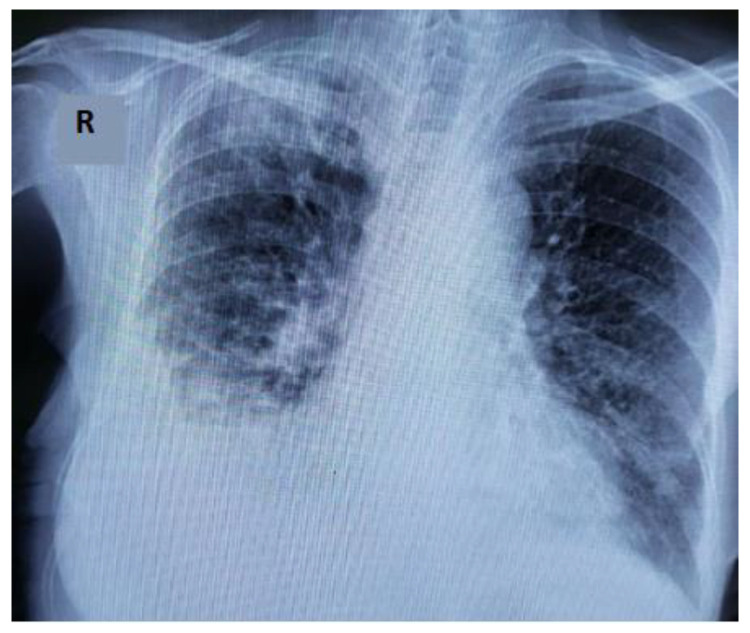
Chest X-ray revealing an increased interstitial infiltration over bilateral lung fields and a juxta-pleural opacity (Hampton hump sign); R-right.

The chest X-ray at the first evaluation in our clinic showed increased interstitial infiltration over bilateral lung fields and a juxta-pleural opacity, interpreted as a Hampton hump sign in the right lung, as is shown in Figure 3.

An emergency pulmonary CT was performed and a diagnosis of lower right pulmonary lobe embolism with additional ischemic tissue changes was made. A Doppler ultrasound of the calf was performed with no evidence of deep/superficial venous thrombosis and an electrocardiogram, as well as an echocardiography, revealed no cardiac lesions. Genetic thrombophilia and antiphospholipid syndrome were excluded as a causality of the procoagulant status, probably incriminating the inflammation as the main cause. The cardiologist prescribed subcutaneous low molecular weight heparin (LMWH) for 7 days, followed by non-vitamin K Antagonist Oral Anticoagulant (NOAC) for 3 months, in association with proton pump inhibitors, according to the current management guidelines and prophylaxis recommendations for pulmonary embolism, with the limitation of lesions [9].

**Figure 4:**
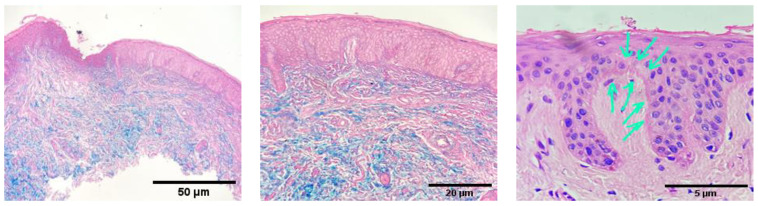
Skin Biopsy revealing inflammatory infiltrate, mucin deposits, basement membrane thickening (arrows).

**Figure 5:**
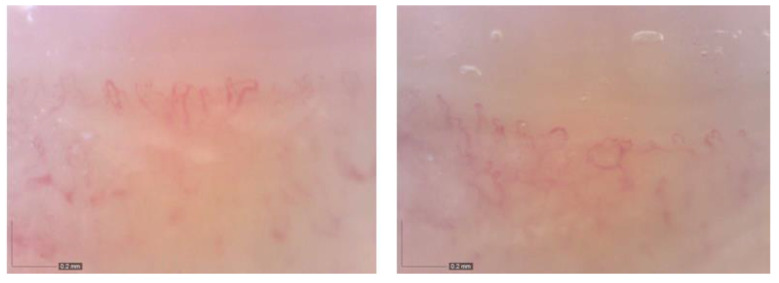
Nailfold video capillaroscopy.

A musculoskeletal ultrasound revealed small amounts of fluid in the flexor tendons and mild effusion in the small joints of the hands, without synovial hypertrophy. The upper and lower endoscopies were normal. A skin biopsy was conducted, and the histopathological exam showed inflammatory infiltrates, mucin deposits, basal membrane thickening, and specific changes compatible with dermatomyositis (Figure 4). The nailfold video capillaroscopy evidenced a decrease in the capillary density, a pattern compatible with myopathies. (Figure 5).

In addition, the myositis antibody panel tests showed strongly positive anti-RO52 and positive anti-MDA5 autoantibodies, Therefore, applying the 2017 EULAR/ACR classification criteria for idiopathic inflammatory myositis, a diagnosis of amyopathic dermatomyositis was sustained [10].

Therefore, the patient was treated with intravenous Methylprednisolone 125 mg daily for 3 days, followed by Prednisone 0.5 mg/kg daily, orally, with a favorable outcome during hospitalization, so she was discharged with Prednisone 20 mg daily (moderate dosage) and immunosuppressive treatment was initiated with Mycophenolate Mofetil 2000 mg daily. Due to the absence of a pneumological evaluation in the context of important pulmonary fibrosis on CT scans, the patient was advised to have a complete respiratory assessment.

At the 1-month follow-up, the clinical condition was significantly improved with the persistence of a mild biological inflammatory syndrome, elevated liver enzymes and increased ferritin level. Between the two hospital admissions, patients were prevented from obtaining pneumological evaluations, due to the SARS-CoV-2 pandemic. We decided to continue the immunosuppression with Mycophenolate Mofetil 2000 mg daily, with weekly tapering of corticosteroids, maintaining the recommendation of pulmonary function evaluation.

**Figure 6:**
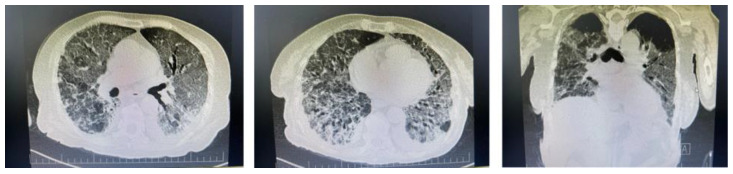
Chest CT scan revealing pulmonary lesions in >90% of total pulmonary architecture.

Two weeks later, the patient tested positive for Severe Acute Respiratory Syndrome Coronavirus 2 (SARS-CoV-2) infection, refusing hospitalization, and decided to stop the MMF, continuing with 10 mg of Prednisone. After another 2 weeks, as a consequence of self-medication, she ended up at the emergency unit, presenting with severe dyspnea, dry cough, tachypnea and a decreased oxygen saturation level (90%). A new chest CT scan was performed, showing diffuse, bilateral honeycombing, ground glass opacities and consolidation affecting more than 50% of the lungs. The procalcitonin level was highly increased 13.1 ng/mL (NV ≤ 0.05 ng/mL) and a septic etiology was suspected without identifying the source of the infection (negative repetitive blood, urine and sputum cultures). Empiric triple antibiotic therapy was administered with a rapidly decreasing dose of procalcitonin. As fast as the infectious status was limited, in the context of the progressive severe interstitial pulmonary fibrosis occupying more than 90% of the parenchymal architecture on chest CT scan re-evaluation at 12 days of admission (Figure 6), the patient received aggressive salvatory immunosuppressive treatment, consisting of intravenous Cyclophosphamide (600 mg/m^2^), Interleukin-1 inhibitor (Anakinra), pulse therapy with Methylprednisolone followed by oral Prednisolone 1 mg/kg daily, to target both etiologies—myositis-related ILD and pulmonary COVID-19.

Although Rituximab is highly recommended for myositis-related ILD, the interdisciplinary team decided to treat according to the actual EULAR guidelines, that strongly suggests postponing or replacing the treatment in COVID-19 positive patients, due to the association of severe pulmonary involvement of SARS-CoV-2 and anti-lymphocyte B treatment [11].

In the therapeutic arsenal, Anakinra was considered as the salvation therapy. Although the studies in recent literature do not attest to the efficacy of Anakinra in DM, it has been successfully used in patients with COVID-19. The inflammatory response to lung injury in SARS-CoV-2 is centrally mediated by IL-1, the main target of Anakinra. The differential diagnosis between the extent of ILD related to COVID-19 and RP-ILD related to DM is very difficult in this case, due to the fact that the patient already had alterations of the lung parenchyma. In aid of treating the ILD related to DM, CYC was administered together with pulse therapy with Methylprednisolone. However, the complex therapy we administered failed to treat the severe respiratory symptoms, the patient’s condition deteriorated, followed by acute respiratory failure and exitus.

The particularity of this difficult to diagnose and treat case consists in the severe rapidly progressive lung involvement due to the myositis pattern, and exacerbated by the association of COVID-19 with pulmonary manifestations, taking into account the limitations of antirheumatic therapy in an infectious context. Even though an aggressive salvatory immunosuppressive treatment was tried, the severity of the ILD was outside of any therapeutical resources.

## 3. Pathogenesis

The exact pathophysiology of dermatomyositis is unknown, however environmental, and viral factors play an important role in genetic susceptibility. MSAs detection could be used to predict the clinical features and disease course of autoimmune myopathy [12]. Initially, there were reported cases of amyopathic dermatomyositis with RP-ILD with poor prognosis in Japanese papers [13,14]. Later, in 2005, Sato et al. described for the first-time a new autoantibody, the CADM 140 antigen, found in 50–70% of CADM cases, and proven to be identical to the anti-MDA5 antibody [8,15]. MDA5 is a viral RNA sensor whose role is to trigger the innate response which increases the production of cytokines and activates the macrophages and helper T cells. One theory for the anti-MDA5 DM mechanism is that the RNA viruses can upregulate MDA5 expression, which can result in excess autoantibodies’ production and initiating cell injuries [5]. Recent studies have shown that the increased MDA5 antibody concentration is associated with disease activity in RP-ILD [16]. Environmental factors play a significant role in this subtype of DM, especially the geographic region; epidemiologic studies show a higher prevalence of anti-MDA CADM in Southeast Asia [17].

## 4. Clinical and Paraclinical Features

MDA5 DM is a heterogeneous entity, but the amyopathic subtype appears to be more frequent in the presence of the anti-MDA5 antibodies. In the absence of muscular symptoms, DM can be a very difficult diagnosis.

Dermatological findings may vary, being divided into two subsets: classical or pathognomonic, and non-specific, less common. The classical skin manifestations appear in approximately 60–70% of patients with MDA5 DM, being represented by Gottron’s papules, Gottron’s sign and heliotrope rash, the cutaneous symptoms included in the 2017 EULAR/ACR classification criteria for idiopathic inflammatory myositis [10,18].

**Figure 7:**
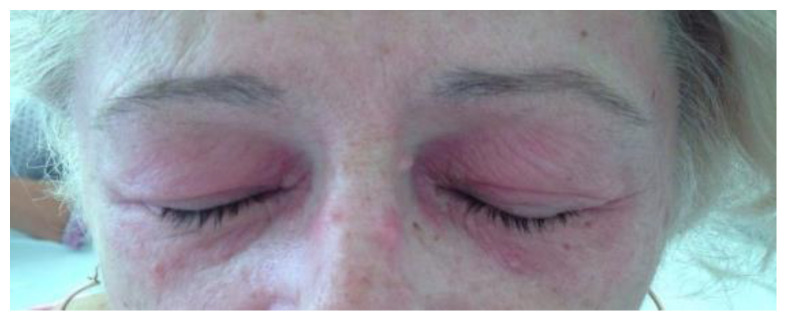
Heliotrope rash in a patient with dermatomyositis.

**Figure 8:**
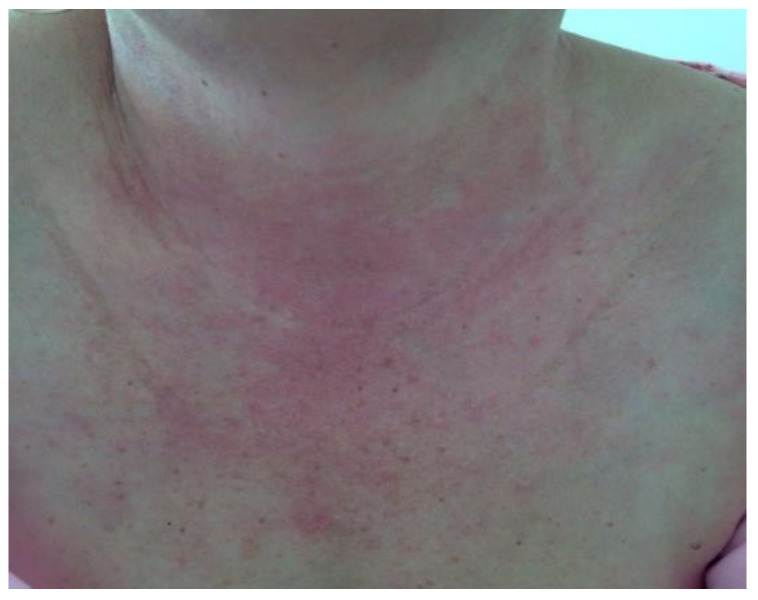
V-sign in a patient with dermatomyositis—erythematous papules located on the anterior thorax.

**Figure 9:**
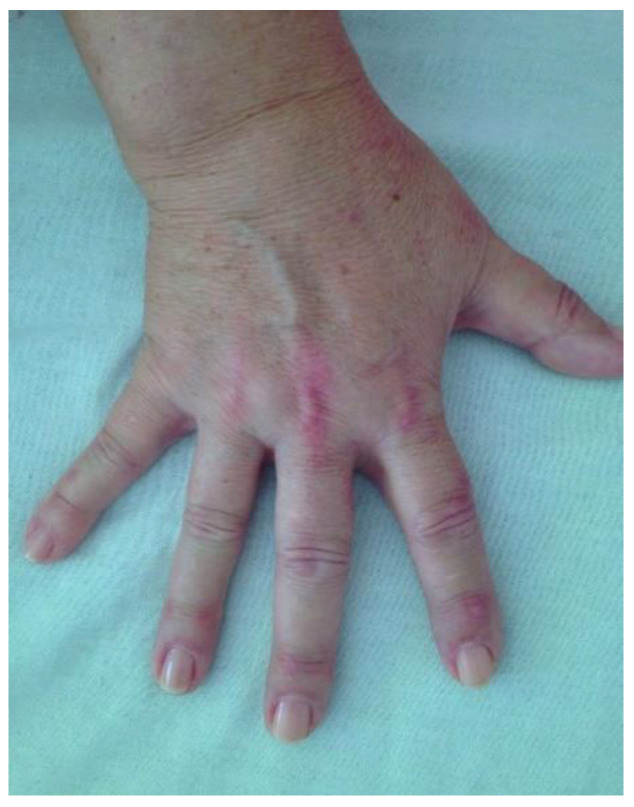
Gottron’s papules and periungual erythema in a patient with dermatomyositis.

Individuals with anti-MDA5 DM can have specific cutaneous manifestations, which is a particularly unique aspect of the condition, and this can lead to a more difficult diagnosis and a delay in treatment. The most common skin findings in anti-MDA5 DM include skin ulceration, mechanic’s hands, palmar papules, heliotrope rash, Gottron’s papules and periungual erythema; the most severe ones being the ulceration which can lead to gangrene, osteomyelitis and eventually to digital amputation [19,20,21]. A supposed mechanism of this lesion is the development of an underlying cutaneous vasculopathy [22,23]. Frequently, the predilection sites for ulcers are overlying the Gottron’s papules in the digital pulp, nailfolds and on the elbows or knees [24]. Narang et al. showed that cutaneous ulcers were strongly associated with anti-MDA5 antibodies, and furthermore that the link between them and the presence of ILD is dependent on anti-MDA5 positivity [25]. Another study revealed that the anti-MDA5 DM patients with normal or lower baseline CK level and ulcerative skin lesions are at higher risk of developing ILD (Figure 7, Figure 8 and Figure 9) [26].

Palmar papules, which appear on the palms and over the interphalangeal and metacarpophalangeal joints on the palmar surface of the fingers, are another distinguishing skin lesion. These findings are frequently inflammatory and painful [24].

A skin biopsy revealed small and medium vascular occlusion with intravascular fibrin deposits and mononuclear inflammatory infiltrate [27]. Other skin biopsy results in anti-MDA5 DM described in the literature are epidermal atrophy, hyperkeratosis, vacuolar interface dermatitis, dermal edema, mucin deposits, basal membrane thickening and CD4+ lymphocytes perivascular infiltrate, but also loss of capillaries and vascular dilatation [28].

In the last decade, nailfold capillaroscopy (NFC) has become an important diagnostic tool for inflammatory myositis, especially in dermatomyositis, due to the vasculopathy involved in this condition. A frequent NFC abnormality is the scleroderma pattern, defined as the presence of mega-capillaries and decreased capillary density with avascular lesions [29]. In a 2015 study, giant capillaries and capillary loss were described, correlated with a short disease duration, while ramified capillaries have been seen in patients with long-term disease [30].

Arthritis and arthralgia are common findings in DM patients, seen in over 50% of patients [5]. There are some similarities with rheumatoid arthritis, the small joints of the hand being frequently affected, typically bilateral and symmetrical [31].

Constitutional symptoms, such as fever, fatigue, and weight loss, are commonly present in patients with DM [5].

Interstitial lung disease is the most serious extra-articular manifestation of dermatomyositis, being the primary cause of morbidity and mortality. There are two forms of ILD based on their onset and evolution: acute/subacute ILD (A/S-ILD); or RP-ILD and chronic ILD (C-ILD) [32]. Many studies have found a correlation between anti-MDA-5 antibodies and the hypo- or amyopathic forms of DM with RP-ILD and acute respiratory failure with a poor prognosis [12,33,34,35]. A meta-analysis showed that the anti-MDA5 positive patients were 20 times more likely to develop RP-ILD compared to seronegative patients [24].

The clinical presentation of patients with ILD includes exercise-induced dyspnea or hypoxemia, fever, cough and deterioration of exercise tolerance [32,36]. According to the international consensus statement on idiopathic pulmonary fibrosis of the American Thoracic Society and the European Respiratory Society, RP-ILD has a rapid progression of the disease in the first 3 months after the onset of symptoms, defined as: symptomatic exacerbation (shortness of breath); increase in parenchymal lesions seen on high resolution computed tomography (HRCT) of the lungs; over 10% decrease in vital capacity or 1.33 kPA decrease in the partial pressure of oxygen (PaO_2_) [33].

In terms of the diagnosis, HRCT is crucial for evaluating and assessing the severity of ILD. In myositis-related ILD, the most common findings are consolidation with ground-glass opacification (GGO), irregular linear opacities, traction bronchiectasis, broncho-vascular thickening and volume loss. The anti-MDA5 antibodies seem to be associated with acute lung injury with a severe organizing pneumonia pattern, that can lead to diffuse alveolar damage and without chronic lesions. Tanizawa et al. conducted various studies on CT imaging in ILD and showed that GGO and consolidation in the lower peripheral lung fields areas with a more diffuse distribution were discovered in anti-MDA5 antibody-positive patients, compared to anti-MDA-5 antibody-negative patients [37]. Kurasawa et al. analyzed 49 cases of A/S-ILD and noticed an association between alveolar opacities with ground glass or reticular opacities and poor prognosis [33].

Another important aspect that needs to be taken into consideration, in the current pandemic context, are the similarities between pulmonary COVID-19 lesions and ILD in the anti-MDA5 DM patients, proper differential diagnosis being mandatory [5,38,39].

Broncho-alveolar lavage reveals alveolitis with mixed cellularity or with increased number of neutrophils, lymphocytes, and eosinophils [32].

Pulmonary function tests are an important tool in assessing the severity of ILD and the respiratory muscle involvement. Restrictive syndrome is frequently noted and involves a decreased total lung capacity (TLC) of under 80% with reduced forced vital capacity (FVC). A decreased diffusing capacity for carbon monoxide (DLCO) generally precedes the TLC and FVC deterioration [32].

The biopsy results of lung tissue from patients with ILD anti-MDA-5 positive showed two distinct patterns: diffuse alveolar damage; and interstitial fibrosis [24].

## 5. Treatment

Although the skin lesions of dermatomyositis can often be resistant to photoprotection, high-protection sunscreen, protective clothes and limited sun exposure are essential for cutaneous manifestations [40]. Pruritus is a common symptom that can influence daily basis activities or sleep patterns, therefore requiring aggressive treatment with topical (Pramoxine, Menthol, Camphor) or oral agents including antihistamines (Doxepin, Hydroxyzine, Cyproheptadine), Amitriptyline or Gabapentin. Topical corticosteroids and topical calcineurin inhibitors are used in the next step of the treatment (e.g., Tacrolimus 0.1%) [41]. Anti-MDA5 positivity is frequently associated with severe skin disease, which is difficult to manage; consequently, most patients need systemic immunosuppressive therapy to achieve remission. Kurtzman et al. suggested intravenous Immunoglobulin (iv Ig) in combination with Mycophenolate Mofetil for refractory cutaneous DM [24]. The pathophysiology of the skin lesions appears to be dominated by vascular injury, for that reason, vasodilators (phosphodiesterase inhibitors—Sildenafil and calcium channel blockers—Nifedipine), endothelin-receptor inhibitor (Bosentan) or drugs that improve peripheral blood flow (Pentoxifylline) may be helpful [5,21].

Patients with myositis-related ILD may benefit from adjuvant therapies, namely annual vaccination against pneumococcus and flu, smoking cessation, oxygen therapy or pulmonary rehabilitation [32].

Pulmonary rehabilitation (PR) includes education and supervised exercise, such as endurance and respiratory training and relaxation techniques. Studies showed that the association between PR and medical treatment improves symptoms, physical capacity, quality of life and patient’s survival [36].

The patients with anti-MDA5 antibodies are more likely to develop severe forms of RP-ILD, having a poor prognosis and survival rate, consequently needing aggressive treatment achieved with combination therapy between corticosteroids and immunosuppressive drugs [42]. A Japanese study that analyzed 29 patients with anti-MDA5-positive ILD, compared combined immunosuppressive therapy with step-up treatment and showed significant improvements regarding survival rates in the combined regimen group (*p* < 0.0001) [43]. A recent systematic review suggested that combination therapy should be the first option in patients with DM-related ILD with MDA5 seropositivity. The usual approach includes glucocorticoids (GC) and calcineurin inhibitors (Cyclosporine A, Tacrolimus) or, alternatively, triple therapy, adding to the previous scheme, intravenous Cyclophosphamide (CYC), or Mycophenolate Mofetil (MM) if CYC is not feasible [44]. GC can be administered orally (Prednisone or Prednisolone) or intravenous (Methylprednisolone) with tapering dosages over several months, dependent on the clinical response [1]. Regarding the calcineurin inhibitors, Cyclosporine A and Tacrolimus showed similar efficacy [32]. In the absence of a response to treatment with GC plus calcineurin inhibitors, adding CYC, MM, Rituximab, Basilixumab or Tofacitinib should be considered [44].

Special attention should be given to CYC’s toxicity and adverse effects. It is known to be teratogenic, embryotoxic and can cause infertility, therefore, if possible, should be avoided in women of childbearing age [45]. In a study that examined 65 patients with rheumatic diseases who were treated with intravenous CYC, the adverse effects reported, in descending order, were: infections, nausea, vomiting, abdominal pain and pancytopenia [46].

Several studies showed the efficacy of Tofacitinib in myositis-associated ILD, concomitantly emphasizing the major risk of viral infections, especially of cytomegalovirus infection (up to 100% of patients) and varicella-zoster virus reactivation (in up to 80% of cases) [47,48].

Numerous studies support the efficacy of Rituximab in anti-MDA5 antibody positive ILD, many patients achieving remission or improvement in pulmonary function and imaging after 3 years of follow-up [35,48,49].

An association between Rituximab and an increased risk of severe COVID-19 was observed [50]. Jyssum et al. analyzed the serological response after two vaccine doses in patients treated with Rituximab in comparison to the general population. A total of 62.1% of those included in the Rituximab group produced no antibodies against SARS-CoV-2, compared to 0.4% of controls. They recommend carefully assessing the risk–benefit ratio and changing the treatment, when possible [51,52].

In the context of a higher risk of severe COVID-19 [53] and lower titers of antibodies against SARS-CoV-2 after vaccination, seen in the patients treated with Rituximab, the European League Against Rheumatism (EULAR) recommends postponing the next cycle of treatment or replacing it, when possible, if the patient contacts the infection [11].

Regarding the COVID-19 vaccine, EULAR suggests initiating it 4 weeks before a new cycle, and to delay Rituximab 2 to 4 weeks after the vaccination [54].

With reference to Anakinra, an interleukin-1 inhibitor, data about its efficacy in myositis related ILD are scarce. Chiapparoli et al. conducted a study that included 15 patients with refractory myositis who were treated with Anakinra for 12 months. Muscle biopsies were performed before and after the treatment. A total of seven patients showed significant clinical response, at the same time, three worsened. The comparative biopsies revealed no differences at the end of the study. In terms of adverse events, the most frequent were infections and rashes at the injection site. Further studies on more patients are required [55,56].

Regarding COVID-19 respiratory syndrome and Anakinra, various studies confirmed its benefits. The inflammatory response to lung injury in SARS-CoV-2 is centrally mediated by IL-1 [57]. An increase in IL-1 concentrations was seen in patients with acute respiratory distress syndrome [58]. Marco Franzetti et al. compared 56 patients with moderate–severe COVID-19, who were treated with Anakinra, to 56 controls. They observed that the 28-day survival rate was higher in the Anakinra group versus those receiving the standard of care treatment (*p* = 0.007). The cumulative risk of death was also lower among these patients (*p* = 0.027). Inflammatory markers, namely C-Reactive protein and Ferritin, showed a significant reduction in the first 7 days of treatment, whereas the lymphocyte count increased notably in patients receiving biologic therapy compared to the controls [59].

## 6. Prognosis

Sugiyama Y. et al. analyzed 116 patients with myositis-related ILD, of whom 43 suffered from clinically amyopathic dermatomyositis. They observed several poor prognostic factors, namely old age, male sex, skin ulcers, seropositivity for MDA-5 antibody, extended lesions in the upper lung fields on HRCT, high initial doses of Prednisone and combination immunosuppressive therapies [60]. The serum ferritin level is a marker of macrophage activation and correlates with a poor prognosis and the severity of the lung involvement. There are other several reports that support the association between anti-MDA-5 antibodies and poor prognosis [32,33,34]. Kurasawa K. et al. consider the titer of these antibodies as a marker of disease activity which can be used to evaluate the efficacy of the treatment [33]. The level of ferritin, C-reactive protein, Ro-52 antibodies and cutaneous vasculopathy also correlate with the severity of the disease and prognosis [5,20,44,61,62,63].

Furthermore, Tanizawa et al. showed that, for patients with DM and ILD, the important prognostic factors are high fever, anti-MDA5 autoantibodies, specific CT shadows (especially lower consolidation) and a ferritin level of over 500 ng/mL, significantly associated with a high 90-day mortality [64]. Moreover, when comparing fatal outcomes for DM individuals with anti-MDA5 and anti-aminoacyl-tRNA synthetase antibodies, the results reported that the alveolar–arterial oxygen gradient and levels of aspartate transaminase and gamma glutamyl transpeptidase are correlated with a poor prognosis among anti-MDA5-positive patients [65].

In a recent study, it was demonstrated that the individuals diagnosed with CADM, having anti-MDA5 positivity and also other myositis-associated antibodies are more likely to have a favorable prognosis, including lower mortality rates, lower incidence of RP-ILD and a better response of ILD to combination therapy, compared to those only having MDA5 positivity [66].

## 7. Conclusions

Anti-MDA5 amyopathic DM is a subtype of myositis, with no muscle involvement and distinctive skin lesions, that are sometimes very discrete. It is associated with an increased risk of developing rapidly progressive interstitial lung disease. Due to a high mortality rate caused by RP-ILD, it is imperative for practitioners to correctly recognize this condition and promptly initiate combined immunosuppressive treatment. The therapeutic approach of our clinical case was limited by the association of SARS-CoV-2 infection and bacterial infection, the causality of impressive pulmonary progression being due to these overlapping entities.

## Data Availability

Not applicable.
